# The effect of acyclovir on the tubular secretion of creatinine *in vitro*

**DOI:** 10.1186/1479-5876-8-139

**Published:** 2010-12-30

**Authors:** Patrina Gunness, Katarina Aleksa, Gideon Koren

**Affiliations:** 1 Division of Clinical Pharmacology and Toxicology, The Hospital for Sick Children, 555 University Avenue, Toronto, Ontario, M5G 1X8, Canada; 2 Graduate Department of Pharmaceutical Sciences, Leslie Dan Faculty of Pharmacy, University of Toronto, 144 College Street, Toronto, Ontario, M5S 3M2, Canada

## Abstract

**Background:**

While generally well tolerated, severe nephrotoxicity has been observed in some children receiving acyclovir. A pronounced elevation in plasma creatinine in the absence of other clinical manifestations of overt nephrotoxicity has been frequently documented. Several drugs have been shown to increase plasma creatinine by inhibiting its renal tubular secretion rather than by decreasing glomerular filtration rate (GFR). Creatinine and acyclovir may be transported by similar tubular transport mechanisms, thus, it is plausible that in some cases, the observed increase in plasma creatinine may be partially due to inhibition of tubular secretion of creatinine, and not solely due to decreased GFR. Our objective was to determine whether acyclovir inhibits the tubular secretion of creatinine.

**Methods:**

Porcine (LLC-PK1) and human (HK-2) renal proximal tubular cell monolayers cultured on microporous membrane filters were exposed to [2-^14^C] creatinine (5 μM) in the absence or presence of quinidine (1E+03 μM), cimetidine (1E+03 μM) or acyclovir (22 - 89 μM) in incubation medium.

**Results:**

Results illustrated that in evident contrast to quinidine, acyclovir did not inhibit creatinine transport in LLC-PK1 and HK-2 cell monolayers.

**Conclusions:**

The results suggest that acyclovir does not affect the renal tubular handling of creatinine, and hence, the pronounced, transient increase in plasma creatinine is due to decreased GFR, and not to a spurious increase in plasma creatinine.

## Background

Acyclovir is an antiviral agent that is commonly used to treat severe viral infections including herpes simplex and varicella zoster, in children [1]. Acyclovir is generally well tolerated [2], however, in some cases, severe nephrotoxicity has been reported [2-8]. Acyclovir - induced nephrotoxicity is typically evidenced by elevated plasma creatinine and urea levels, the occurrence of abnormal urine sediments or acute renal failure [2-5,7,8].

Crystalluria leading to obstructive nephropathy is widely believed to be the mechanism of acyclovir - induced nephrotoxicity [9]. However, there are several documented cases of acyclovir - induced nephrotoxicity in the absence of crystalluria [7,8,10]; suggesting that acyclovir induces direct insult to tubular cells. Recently, we provided the first *in vitro *experimental evidence which supports existing clinical evidence of direct renal tubular damage induced by acyclovir [11].

A systematic review of the literature reveals a pronounced, transient elevation (up to 9 fold in some cases) of plasma creatinine levels in children, often without any other clinical evidence of overt nephrotoxicity (Table 1). Similar to the cases described in Table 1; a marked, transient increase in plasma creatinine levels has been observed in some patients who received the non-nephrotoxic drugs, cimetidine [12-16], trimethoprim [17-19], pyrimethamine [20], dronedarone [21] and salicylates [22].

**Table 1:** Cases of elevated plasma creatinine levels in children who received intravenous acyclovir

Patient	Magnitude of increase in plasma creatinine (from baseline)	Relevant clinical details	References
1 child	**5 fold **increase within 2 days	Creatinine returned to normal in 4 days Elevated urea No other pathology reported	[4]
10 children	transient elevation	No further impairment reported	[2]
3 children	**4 fold **increase within 4 days	Mild reduction in urine output Creatinine returned to normal 1 week following acyclovir discontinuation	[3]
1 child	**2 fold **increase within 6 days	Creatinine continued to increase following acyclovir discontinuation. Creatinine returned to normal within 1 week Elevated urea Mild proteinuria	[7]
3 children	**9 fold **increase within 2 to 3 days	High urea Urinary α_1_-microglobulin and albumin Creatinine returned to normal in 3 - 9 days	[8]
1 child	**3 fold **increase within 4 days	No other information provided	[5]

Creatinine, a commonly used biomarker that is used to assess renal function, is eliminated by the kidney via both glomerular filtration and tubular secretion [23]. The mechanisms underlying the renal tubular transport of creatinine has not been fully elucidated. As explained by Urakami and colleagues [24], both acid and base secreting mechanisms may play a role in the renal tubular transport of creatinine [13-15,17-22,25-27]. Hence, some drugs may share similar renal tubular transport mechanisms with creatinine. Drugs that share transport mechanisms with creatinine may compete with it for tubular transport, and subsequently inhibit creatinine secretion to result in a ungenuine elevation of plasma creatinine that may not be due to decreased glomerular filtrate rate (GFR). Cimetidine [12-16], trimethoprim [17-19], pyrimethamine [20], dronedarone [21] and salicylates [22] are examples of drugs that share similar renal tubular transport mechanisms with creatinine and induce spurious increases in plasma creatinine by competing with and subsequently inhibiting its secretion.

Similar to creatinine, both acid and base secreting pathways may be involved in the renal tubular transport of acyclovir [28]. Additionally, it is likely that creatinine [24-26] and acyclovir [28] may be transported by similar organic anion transporters (OAT) and organic cation transporters (OCT). Therefore, it is plausible that acyclovir may compete with and successively inhibit renal secretion of creatinine, resulting in elevations in plasma creatinine that may be disproportional to the degree of renal dysfunction.

Employing plasma creatinine levels to estimate GFR, results from previous studies [29,30] have illustrated that acyclovir - induced nephrotoxicity induces a significant reduction in GFR in children. However, based on: (1) the cases presented in Table 1, (2) the awareness that several non-nephrotoxic drugs are known to induce transient increases in plasma creatinine [12-22] and (3) the knowledge that acyclovir and creatinine may share similar renal tubular transport mechanisms; we hypothesized that the pronounced, transient increase in plasma creatinine levels observed in some patients may be partially due to the inhibition of renal tubular secretion of creatinine by acyclovir, and not entirely the result of decreased GFR. To the best of our knowledge, the effect of acyclovir on the renal tubular secretion of creatinine *in vitro *has not been previously evaluated. Thus, the objective of the study was to determine whether acyclovir inhibits the renal tubular secretion of creatinine. It is important to determine whether acyclovir inhibits the tubular transport of creatinine, because if this is the case, then in addition to creatinine, other biomarkers should always be employed to assess renal function in patients receiving acyclovir treatment.

In the present study we were specifically interested in determining the possible interaction between creatinine and acyclovir during renal tubular transport by the OCT pathway. The porcine renal tubular cell line, LLC-PK1, has been used as an *in vitro *renal tubular model in a vast array of transepithelial transport studies. Furthermore, the LLC-PK1 cells are an appropriate *in vitro *model for specifically studying renal tubular transport of organic cations because they are known to possess functional OCTs [31-33]. However, although the LLC-PK1 cells retain similar physiological and biochemical properties compared to human renal proximal tubular cells [34], interspecies differences in drug disposition exists [35-37]. Hence, the use of a human renal proximal tubular cell line, such as the HK-2 cell line, would be a more suitable *in vitro *model to study the mechanisms of renal tubular drug transport in humans. Porcine LLC-PK1 and human HK-2 cells were employed in our transepithelial transport studies.

## Methods

### Cell culture

The LLC-PK1 cells (American Type Culture Collection (ATCC), USA) were cultured in growth medium which consisted of Minimum Essential Medium (MEM) alpha modified (Fisher Scientific, Canada), supplemented with 2 mM L-glutamine, 100 units/mL penicillin, 100 μg streptomycin and 10% (v/v) fetal bovine serum (Invitrogen Canada Inc., Canada). The HK-2 cells (ATCC) were cultured in growth medium which consisted of Keratinocyte-Serum Free Medium, supplemented with human recombinant epidermal growth factor 1-53 (5 ng/mL) and bovine pituitary extract (0.05 mg/mL) (Invitrogen Canada Inc.) The LLC-PK1 and HK-2 cells were maintained at 37°C in a sterile, humidified atmosphere of 5% CO_2 _and 95% O_2_.

### Transepithelial transport studies

The transepithelial transport studies were conducted as outlined by Urakami et al. [33] with modifications. The LLC-PK1 and HK-2 cells were seeded at densities of 4.5E+05 cells/0.9 cm^2 ^and 5.0E+05 cells/0.9 cm^2^, respectively, on microporous membrane filter inserts (3 μm pore size, 0.9 cm^2 ^growth area) that were placed inside cell culture chambers (VWR International, Canada). A consistent (1 mL) volume of growth or incubation medium (containing no substrates, radiolabeled or non-radiolabeled substrates) was placed in the apical and basolateral compartments of the cell culture chambers during culturing of the cells or during all transport experiments. The LLC-PK1 and HK-2 cell monolayers used for transport studies were cultured in growth medium for 6 and 3 days, respectively, after seeding. All transepithelial transport studies were conducted on confluent cell monolayers.

At the time of commencement of the transport experiments, the growth medium from the cell culture chamber was removed and both sides of the cell monolayers were washed twice with incubation medium (145 mM NaCl, 3 mM KCl, 1 mM CaCl_2_, 0.5 mM MgCl_2_, 5 mM D-glucose and 5 mM HEPES (pH 7.4)). Incubation medium was used for all transport experiments. Cell monolayers were incubated with medium for 10 minutes. Following the 10 minute incubation period, the medium was removed and the cell monolayers were incubated with medium as follows: the medium added to the basolateral compartment of the cell culture chamber contained respective radiolabeled and non-radiolabeled substrates and the medium added to the apical compartment of the cell culture chamber contained neither radiolabeled nor non-radiolabeled substrates. The radiolabeled and non-radiolabeled substrates used in the transport studies are outlined below.

The transepithelial transport (basolateral-to-apical) of radiolabeled substrates across the cell monolayers was assessed at specific intervals (LLC-PK1: 0, 15, 30, 45 and 60 minutes; HK-2: 0, 7.5, 15, 22.5 and 30 minutes) over 60 and 30 minutes, respectively. Studies were conducted over different duration of times in LLC-PK1 and HK-2 cells due to differences in the integrity of the cell monolayers. The paracellular flux (basolateral-to-apical) of D-[1-^3^H(N)] mannitol (PerkinElmer, Canada) across the cell monolayers was used to assess the integrity of cell monolayers. *A priori *decision was made to eliminate the results from any cell monolayers where the paracellular flux of D-[1-^3^H(N)] mannitol across LLC-PK1 or HK-2 cell monolayers was greater than 5% over the respective experimental period.

The transport of radiolabeled substrates was assessed by measuring the radioactivity of 50 μL aliquots of medium that were sampled from the apical and basolateral compartments of the cell culture chamber, at the aforementioned specified time intervals for the respective cell line. Radioactivity was measured as disintegrations per minutes (DPM) using a LS 6500 liquid scintillation (Beckman Coulter Canada Inc., Canada).

#### Tetraethylammonium (TEA) transport across cell monolayers

In order to determine whether the LLC-PK1 and HK-2 cells used in the present studies possessed functional organic cation transporters; TEA transport across cell monolayers was assessed. The TEA is a classical organic cation substrate for OCTs [31,32,38]. The transport of TEA across LLC-PK1 and HK-2 cell monolayers was assessed in the presence and absence of the known inhibitor of organic cation transport [24,31-33], quinidine (Sigma-Aldrich Canada Ltd., Canada). Cell monolayers were incubated with medium (containing [ethyl-1-^14^C] TEA (5 μM) (American Radiolabeled Chemicals Inc., USA) in the presence or absence of quinidine (1E+03 μM). The transport of TEA was assessed as described above.

#### Acyclovir transport across cell monolayers

The transport of acyclovir across LLC-PK1 or HK-2 cell monolayers was assessed in the presence or absence of quinidine. Cell monolayers were incubated with medium (containing [8-^14^C] acyclovir (5E-05 μM) (American Radiolabeled Chemicals Inc.)) in the presence or absence of quinidine (1E+03 μM). The transport of acyclovir was assessed as described above.

#### The effect of acyclovir on creatinine transport across cell monolayers

The transport of creatinine was assessed across LLC-PK1 or HK-2 cell monolayers in the presence or absence of acyclovir. Cell monolayers were incubated with medium (containing [2-^14^C] creatinine (5 μM) (American Radiolabeled Chemicals Inc.)) in the presence or absence of quinidine (1E+03 μM), cimetidine (1E+03 μM) (Sigma-Aldrich Canada Ltd.) or acyclovir (22 to 89 μM) (Pharmacy at the Hospital for Sick Children, Canada). The acyclovir concentrations used in the experiments are representative of concentrations of acyclovir that are found in the plasma and hence, are the concentrations which creatinine may encounter in plasma.

### Statistical analyses

Statistical analyses were performed using ANOVA followed by Tukey's HSD post hoc tests. Statistical analyses were performed on substrate radioactivity (DPM) data. Data are presented as the mean ± standard error (SE) from 3 cell monolayer experiments. Data were considered statistically significant if p < 0.05.

## Results

### TEA transport across LLC-PK1 and HK-2 cell monolayers

**Figure 1:**
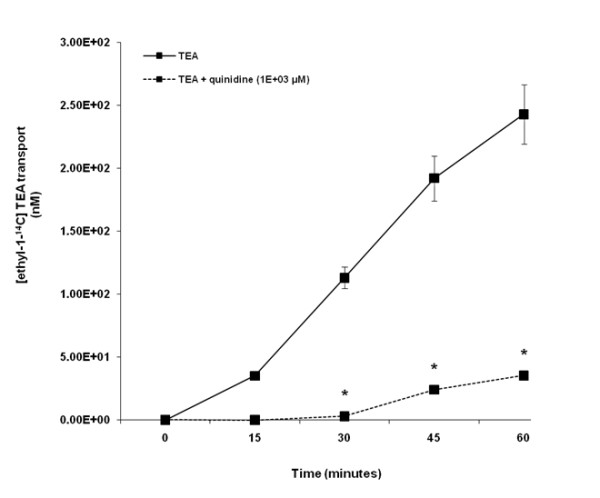
**Tetraethylammonium (TEA) transport across porcine renal proximal tubular cell (LLC-PK1) monolayers**. The transport (basolateral-to-apical) of TEA was assessed in LLC-PK1 cells monolayers. Cell monolayers were exposed to [ethyl-1-^14^C] TEA (5 μM) in the presence or absence of quinidine (1E+03 μM) for 60 minutes. The transport of TEA was assessed by measuring the appearance of [ethyl-1-^14^C] TEA radioactivity in the apical compartment at specific time intervals (0, 15, 30, 45 and 60 minutes) for 60 minutes. Radioactivity was measured as disintegrations per minute (DPM). The TEA transport is expressed as the concentration of [ethyl-1-^14^C] TEA in the apical compartment. Results are presented as the mean (±standard error (SE)) from 3 cell monolayer experiments. * p < 0.05, compared to [ethyl-1-^14^C] TEA radioactivity in the apical compartment in the absence of quinidine.

The TEA was transported across LLC-PK1 cell monolayers in a time - dependent manner over the experimental study period (Figure 1). The results illustrate that there was a significant (p < 0.05) decrease in the concentration of [ethyl-^14^C] TEA in the apical compartment in the presence of quinidine at 30, 45 and 60 minutes.

**Figure 2:**
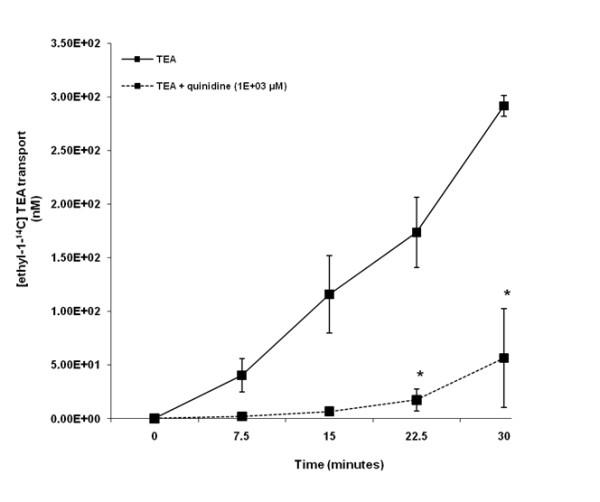
**Tetraethylammonium (TEA) transport across human renal proximal tubular cell (HK-2) monolayers**. The transport (basolateral-to-apical) of TEA was assessed in HK-2 cells monolayers. Cell monolayers were exposed to [ethyl-1-^14^C] TEA (5 μM) in the presence or absence of quinidine (1E+03 μM) for 30 minutes. The transport of TEA was assessed by measuring the appearance of [ethyl-1-^14^C] TEA radioactivity in the apical compartment at specific time intervals (0, 7.5, 15, 22.5 and 30 minutes) for 30 minutes. Radioactivity was measured as disintegrations per minute (DPM). The TEA transport is expressed as the concentration of [ethyl-1-^14^C] TEA in the apical compartment. Results are presented as the mean (±standard error (SE)) from 3 cell monolayer experiments. * p < 0.05, compared to [ethyl-1-^14^C] TEA radioactivity in the apical compartment in the absence of quinidine.

Our results illustrate that TEA was transported across HK-2 cell monolayers in a time - dependent manner over the experimental period (Figure 2). The concentration of [ethyl-^14^C] TEA in the apical compartment was significantly (p < 0.05) decreased in the presence of quinidine at 22.5 and 30 minutes.

### Acyclovir transport across LLC-PK1 and HK-2 cell monolayers

**Figure 3:**
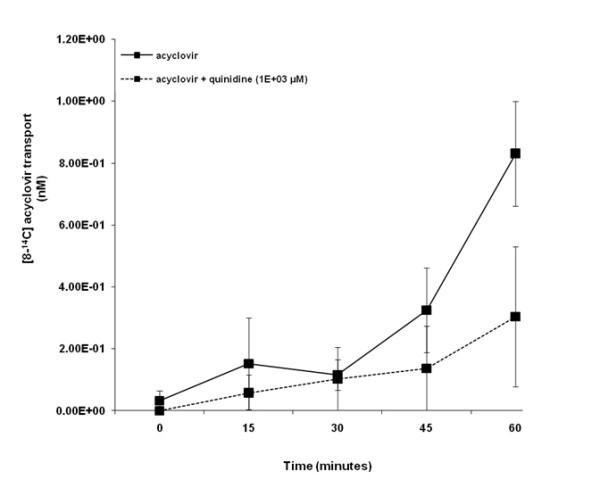
**Acyclovir transport across porcine renal proximal tubular cell (LLC-PK1) monolayers**. The transport (basolateral-to-apical) of acyclovir was assessed in LLC-PK1 cells monolayers. Cell monolayers were exposed to [8-^14^C] acyclovir (5E-02 μM) in the presence or absence of quinidine (1E+03 μM) for 60 minutes. The transport of acyclovir was assessed by measuring the appearance of [8-^14^C] acyclovir radioactivity in the apical compartment at specific time intervals (0, 15, 30, 45 and 60 minutes) for 60 minutes. Radioactivity was measured as disintegrations per minute (DPM). Acyclovir transport is expressed as the concentration of [8-^14^C] acyclovir in the apical compartment. Results are presented as the mean (±standard error (SE)) from 3 cell monolayer experiments.

Acyclovir appeared to be transported across LLC-PK1 cell monolayers in a time - dependent manner from 30 to 60 minutes (Figure 3). There was a trend of decreased concentration of [8-^14^C] acyclovir in the apical compartment in the presence of quinidine over the experimental study period. Acyclovir transport was not significantly (p > 0.05) inhibited in the presence of quinidine.

**Figure 4:**
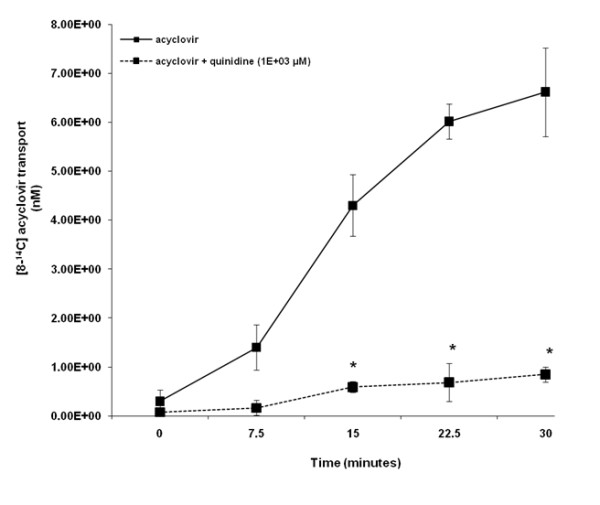
**Acyclovir transport across human renal proximal tubular cell (HK-2) monolayers**. The transport (basolateral-to-apical) of acyclovir was assessed in HK-2 cells monolayers. Cell monolayers were exposed to [8-^14^C] acyclovir (5E-02 μM) in the presence or absence of quinidine (1E+03 μM) for 30 minutes. The transport of acyclovir was assessed by measuring the appearance of [8-^14^C] acyclovir radioactivity in the apical compartment at specific time intervals (0, 7.5, 15, 22.5 and 30 minutes) for 30 minutes. Radioactivity was measured as disintegrations per minute (DPM). Acyclovir transport is expressed as the concentration of [8-^14^C] acyclovir in the apical compartment. Results are presented as the mean (±standard error (SE)) from 3 cell monolayer experiments. * p < 0.05, compared to [8-^14^C] acyclovir radioactivity in the apical compartment in the absence of quinidine.

Acyclovir was transported across HK-2 cell monolayers in a time - dependent manner over the experimental study period (Figure 4). Results illustrate that the concentration of [8-^14^C] acyclovir in the apical compartment was significantly (p < 0.05) decreased in the presence of quinidine at 15, 22.5 and 30 minutes.

### The effect of acyclovir on creatinine transport across LLC-PK1 and HK-2 cell monolayers

**Figure 5:**
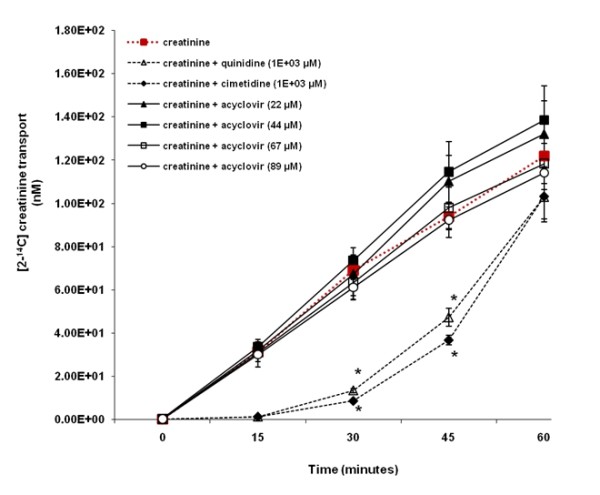
**The effect of acyclovir on creatinine transport across porcine renal proximal tubular cell (LLC-PK1) monolayers**. The transport (basolateral-to-apical direction) of creatinine was assessed in LLC-PK1 cells monolayers. Cell monolayers were exposed to [2-^14^C] creatinine (5 μM) in the presence or absence of quinidine (1E+03 μM), cimetidine (1E+03 μM) or acyclovir (22 to 89 μM) for 60 minutes. The transport of creatinine was assessed by measuring the appearance of [2-^14^C] creatinine radioactivity in the apical compartment at specific time intervals (0, 15, 30, 45 and 60 minutes) for 60 minutes. Radioactivity was measured as disintegrations per minute (DPM). Creatinine transport is expressed as the concentration of [2-^14^C] creatinine in the apical compartment. Results are presented as the mean (±standard error (SE)) from 3 cell monolayer experiments. * p < 0.05, compared to [2-^14^C] creatinine radioactivity in the apical compartment in the absence of quinidine, cimetidine or acyclovir.

Figure 5 illustrates that in contrast to quinidine and cimetidine, acyclovir (22 to 89 μM) did not inhibit creatinine transport across LLC-PK1 cell monolayers. The concentration of [2-^14^C] creatinine in the apical compartment over the experimental study period was similar between cell monolayers exposed to creatinine in the presence or absence of acyclovir (22 to 89 μM). In contrast, there was a decrease in the concentration of [2-^14^C] creatinine in the apical compartment in the presence of quinidine or cimetidine, compared to the concentration of [2-^14^C] creatinine in the apical compartment in the absence of quinidine or cimetidine. Creatinine transport was significantly (p < 0.05) inhibited in the presence of quinidine or cimetidine at 30 and 45 minutes.

**Figure 6:**
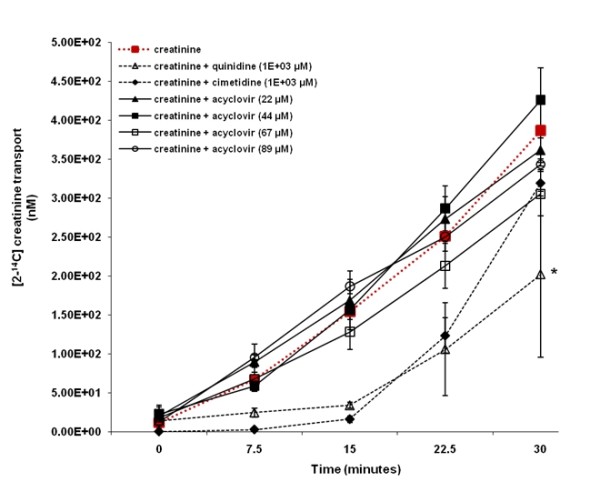
**The effect of acyclovir on creatinine transport across human renal proximal tubular cell (HK-2) monolayers**. The transport (basolateral-to-apical) of creatinine was assessed in HK-2 cells monolayers. Cell monolayers were exposed to [2-^14^C] creatinine (5 μM) in the presence or absence of quinidine (1E+03 μM), cimetidine (1E+03 μM) or acyclovir (22 to 89 μM) for 30 minutes. The transport of creatinine was assessed by measuring the appearance of [2-^14^C] creatinine radioactivity in the apical compartment at specific time intervals (0, 7.5, 15, 22.5 and 30 minutes) for 30 minutes. Radioactivity was measured as disintegrations per minute (DPM). Creatinine transport is expressed as the concentration of [2-^14^C] creatinine in the apical compartment. Results are presented as the mean (±standard error (SE)) from 3 cell monolayer experiments. * p < 0.05, compared to [2-^14^C] creatinine radioactivity in the apical compartment in the absence of quinidine, cimetidine or acyclovir.

Figure 6 illustrates that in contrast to quinidine, acyclovir (22 to 89 μM) did not inhibit creatinine transport across HK-2 cell monolayers. The concentration of [2-^14^C] creatinine in the apical compartment over the experimental study period was similar between cell monolayers exposed to creatinine in the presence or absence of acyclovir (22 to 89 μM). In contrast, the concentration of [2-^14^C] creatinine was decreased in the apical compartment in the presence of quinidine, compared to the concentration of [2-^14^C] creatinine in the apical compartment in the absence of quinidine. Creatinine transport was significantly (p < 0.05) inhibited in the presence of quinidine at 30 minutes. The concentration of [2-^14^C] creatinine appeared to be decreased in the apical compartment in presence of cimetidine, compared to the concentration of [2-^14^C] creatinine in the apical compartment in the absence of cimetidine.

## Discussion

The objective of our study was to determine whether acyclovir inhibits creatinine transport. The LLC-PK1 and HK-2 cell lines were employed as our *in vitro *models. The results suggest that LLC-PK1 (Figure 1) and HK-2 (Figure 2) cells possess functional OCTs, thereby making them appropriate models to study the renal tubular transport of organic cations such as creatinine and acyclovir. In contrast to LLC-PK1 cells, the presence of functional OCTs in HK-2 cells has not been previously reported. Hence, our study is the first to report that HK-2 cells possess functional OCTs, thereby making them an invaluable *in vitro *model to study the renal tubular transport of organic cations in humans.

Importantly, in contrast to quinidine (LLC-PK1 and HK-2) (Figures 5 and 6) or cimetidine (LLC-PK1) (Figure 5), acyclovir did not inhibit creatinine transport across both types of cell monolayers; suggesting that acyclovir does not affect the renal tubular handling of creatinine. As previously explained; (1) the marked, transient increase in plasma creatinine observed in some patients who received acyclovir (Table 1) is similar to that observed in some patients who received non-nephrotoxic drugs that share similar renal tubular transport with creatinine and hence compete with and subsequently inhibit creatinine secretion [12-22] and (2) acyclovir may share similar renal tubular transport mechanisms with creatinine [24-26,28]. Hence, if this is the case, it is possible that our results illustrate that acyclovir did not inhibit the tubular transport of creatinine for the following reasons:

First, as reviewed by Andreev et al. [39], some drugs, such as phenacemide and vitamin D derivatives induce a marked, transient increase in plasma creatinine in the absence of other significant signs of renal impairment by other less well understood mechanisms, including interference with the Jaffé-based assay for creatinine measurement and modification of the production rate and release of creatinine, respectively. Thus, acyclovir may affect plasma creatinine levels by a yet unknown mechanism(s).

Second, based on our results, it can be argued that acyclovir did not inhibit creatinine transport across LLC-PK1 cell monolayers because in contrast to creatinine (Figure 5), the OCT pathway in the LLC-PK1 cells did not appear to play a significant role in acyclovir transport (Figure 3), and hence acyclovir was unlikely to compete with and subsequently inhibit creatinine transport via the OCT pathway present in the cells. Furthermore interspecies differences in drug disposition[35,36] and protein expression [40] for instance, may provide an explanation for the lack of inhibition of creatinine transport by acyclovir in LLC-PK1 cells. For example, the degree of amino acid sequence similarity between porcine OCT1 (pOCT1) and hOCT1 is approximately 78% [41], while porcine OCT2 (pOCT2) and hOCT2 share approximately 86% amino acid sequence homology [42].

However, in contrast to the results obtained in LLC-PK1 cells, the OCT pathway in human HK-2 cells played a significant role in both acyclovir (Figure 4) and creatinine transport (Figure 6), yet similar to the results obtained in LLC-PK1 cells, acyclovir did not inhibit creatinine transport in human HK-2 cells. The results from previous studies suggest that the OCTs may mediate the renal tubular transport of both creatinine [24,25] and acyclovir [28]. However, while OCT2 appears to be primarily responsible for creatinine transport [24,25], it appears that OCT1 may be predominantly accountable for acyclovir transport [28]. Reviewed by Dresser et al. [43], OCT1 and OCT2 are both located in the human kidney, therefore it is possible that renal secretion of creatinine and acyclovir may be mediated by different OCTs; OCT2 and OCT1, respectively. Thus, acyclovir may not impede creatinine tubular transport *in vitro *and possibly *in vivo*, in humans as well.

The knowledge that OCT1, rather than OCT2, mediate acyclovir transport may also provide an explanation for the insignificant transport of acyclovir across LLC-PK1 cells (Figure 3). In contrast to OCT2 [44], OCT1 has not been specifically identified in LLC-PK1 cells. The LLC-PK1 cells may lack or have reduced expression of OCT1. Therefore, LLC-PK1 cells may be unable to transport acyclovir via their existing OCT system, and hence may be an inappropriate model to examine acyclovir transport via the same. Furthermore, if the plausible lack of or reduced OCT1 expression in LLC-PK1 cells resulted in the absence of significant acyclovir transport across the cell monolayers (Figure 3), then the results provide additional support for the postulation that acyclovir and creatinine may be transported via different OCTs.

Third, we employed *in vitro *models in our studies. Although *in vitro *models are widely used in pharmacology and toxicology studies to address questions at both the cellular and molecular level, there are several major disadvantages of *in vitro *models that limit their ability to accurately predict responses *in vivo *[37,45]. Major disadvantages include disruption of cellular structural integrity and intercellular relationships, the production of artifactual drug binding sites that does not normally exist *in vivo*, differences between *in vitro *and *in vivo *drug pharmacokinetics and altered protein expression [37]. Therefore, the transport of creatinine and/or acyclovir *in vitro *may be altered from its transport *in vivo*, in humans.

In our study, we investigated the possible interaction between creatinine and acyclovir at the OCT pathway. However, it is also possible that the interaction between creatinine and acyclovir may be occurring at the OAT pathway, rather than at the OCT pathway. Results from studies suggest that the OAT system may play a fundamental role in both creatinine [22,26,27] and acyclovir [28] transport. The LLC-PK1 cells do not possess OATs [46,47], and therefore are an inappropriate *in vitro *model to study the possible interaction between creatinine and acyclovir at the OAT pathway. The expression of functional OATs in HK-2 cells is currently unknown and we did not determine the same in our study. However, if functional OATs are expressed in HK-2 cells, and both creatinine and acyclovir were significantly transported by the same OAT(s), then, in the presence of acyclovir, decreased creatinine transport across the cell monolayers would have likely been observed. Alternatively, as suggested for OCTs, creatinine and acyclovir may have been transported by different OATs expressed in the HK-2 cells, such that acyclovir did not hinder creatinine transport via the OAT pathway.

## Conclusions

Engaging both animal (LLC-PK1) and human (HK-2) cell models, we illustrated that acyclovir did not inhibit creatinine transport. Taken together, the results suggest that acyclovir does not affect the renal tubular transport of creatinine, *in vitro *and possibly, *in vivo*, in humans as well. Therefore, the pronounced, transient elevation in plasma creatinine observed in some children may be solely due to decreased GFR as a result of renal dysfunction induced by acyclovir, and not due to a spurious acyclovir-creatinine interaction on the tubular level.

## Competing interests

The authors declare that they have no competing interests.

## Authors' contributions

All authors have read and approved the final manuscript submitted to the journal. All authors were involved in the conception and design of the experiments. PG performed all experiments and prepared the draft of the manuscript. All authors participated in editing the manuscript. PG prepared the final manuscript for submission to the journal.
